# Breastfed and mixed fed infants who do not consume infant cereal are at risk for inadequate iron intake:data from the feeding infants and toddlers study 2016, a cross-sectional survey

**DOI:** 10.1186/s12887-022-03104-9

**Published:** 2022-04-22

**Authors:** Kristen Finn, Sarah Quick, Andrea Anater, Joel Hampton, Brian Kineman, William Klish

**Affiliations:** 1 Nestlé Nutrition, Medical, Scientific, and Regulatory Affairs Unit, 1812 N. Moore St, Arlington, VA 2209 USA; 2 DSM Nutritional Products, Parsippany, NJ USA; 3 Nestlé Product Technology and Development Center, Singen, Germany; 4 RTI International, Durham, NC USA; 5 Baylor College of Medicine, Houston, TX USA

**Keywords:** Nutrition, Iron, Breastfeeding, Complementary Feeding, Infant Cereal

## Abstract

**Background:**

According to the Feeding Infants and Toddlers Study (FITS), the percentage of older infants consuming infant cereal has declined from 72% of 6–11.9 month old infants in 2002 to 52% in 2016. This is especially concerning for breastfed and mixed fed infants because of their increased need for dietary sources of iron. This study explored the association between infant cereal consumption and nutrient intakes among breastfed and mixed fed infants.

**Methods:**

FITS 2016 is the largest cross-sectional survey of food and nutrient intakes among caregivers of children less than 4 years old in the United States. For this analysis, we evaluated 24 h dietary recalls for infants 6–11.9 months who were either breastfed (no infant formula provided, *n* = 296) or mixed fed (breastmilk and infant formula provided, *n* = 102). Infants were further categorized as infant cereal consumers or non-consumers. Nutrient intakes were compared with Adequate Intakes or Estimated Average Requirements when available. Differences between cereal consumers and non-consumers were calculated using unpaired T-tests.

**Results:**

Significantly fewer breastfed cereal consumers had intakes below the Estimated Average Requirement for iron (19% vs. 96%) and zinc (61% vs. 16%, *p* < 0.0001). Additionally, significantly more breastfed cereal consumers had intakes above the Adequate Intake level for 12 other nutrients compared to non-consumers. Among mixed fed infants, significantly fewer cereal consumers had intakes below the Estimated Average Requirement for iron compared to non-consumers (5% vs. 70%), but differences in other nutrients were not observed.

**Conclusions:**

Almost all (96%) of the breastfed infants who did not consume infant cereal had inadequate iron intakes. Even among mixed fed infants, significantly fewer infant cereal consumers had inadequate iron intakes compared to non-cereal consumers. Infant cereal is an important source of iron and other key nutrients, especially for infants receiving breastmilk.

## Introduction

Consumption of fortified infant cereal among infants in the US has dramatically declined since 2002 [1, 2]. This is concerning as infant cereal is the top complementary food source of iron and nine other essential vitamins and minerals for infants [3]. According to the Feeding Infants and Toddlers Study (FITS) which is a large US cross-sectional dietary intake study, the number of caregivers who reported feeding infant cereal to their 6–11.9 month old infants decreased from 76% in 2002, to 52% in 2016 [1]. In the same time frame, the number of 6–11.9 month old infants with iron intakes below the Estimated Average Requirement (EAR) increased from 7.5% in 2002 to 18% in 2016 [4–6].

It is well known that iron deficiency anemia in early life can cause deleterious and irreversible effects on behavioral, motor, and mental development [7–9]. Historically, in the US, iron deficiency anemia was common among infants and toddlers [10, 11]. The fortification of infant cereal was a key factor in a multi-pronged initiative to combat iron deficiency beginning in the 1970’s, which also included the initiation of the Supplemental Nutrition Program for Women, Infants, and Children (WIC) which provides infant cereal and iron fortified infant formula as well as other nutrient rich foods to income eligible participants [11]. By the mid-1980’s, the incidence of iron deficiency anemia among infants and young children had dropped drastically [11, 12]. Still, iron deficiency anemia continues to impact young children in the US, especially among low income and minority groups [13]. The most recent estimate from the National Health and Nutrition Examination Surveys (NHANES) 2002–2010 found that 15.1% of one to 2 year old children were iron deficient [13]. It is unknown if the decline in infant cereal consumption has had an impact on the incidence of iron deficiency anemia because there is no national surveillance among infants and no current statistics among toddlers [14]. As positive strides are achieved in increasing the prevalence of breastfeeding in the US, the inclusion of iron-rich complementary food sources become even more important [15, 16].

Currently, the American Academy of Pediatrics (AAP) recommends exclusive breastfeeding for around the first 6 months of life, followed by continued breastfeeding supplemented with two servings of iron and zinc rich foods (meat and/or fortified infant cereal) per day which is consistent with several other pediatric nutrition guidelines [15, 17]. In their recently publicized scientific report, the US Dietary Guidelines Advisory Committee recommended iron fortified cereal or similar products for infants aged 6–12 months receiving breastmilk for adequate iron intake [18].

Previous analyses of FITS 2008 data evaluated the differences in iron intakes among infants who did and did not consume infant cereal [3]. Infant cereal non-consumers had iron intakes 50–60% lower than cereal consumers [3]. More non-consumers had iron intakes below the EAR for iron compared to cereal consumers (47% vs 7% of 6–8.9 month olds and 33% vs. 7% of 9–11.9 month olds, respectively) [3]. In addition, more infants who did not consume infant cereal were breastfed compared to cereal consumers (64% vs. 45% of 6–8.9 month old infants and 54% vs. 36% of 9–11.9 month old infants) [3].

The purpose of this study was to compare the adequacy of nutrient intakes between infant cereal consumers and non-consumers among breastfed and mixed fed infants. We hypothesized that breastfed and mixed fed infants who consumed infant cereal (infant cereal consumers) would be more likely to have sufficient nutrient intakes compared to those who did not consume infant cereal (non-consumers).

## Methods

A detailed description of the FITS 2016 study design and methods are available in a separate publication [19]. The FITS 2016 was a follow up to surveys conducted in 2002 and 2008 [20, 21]. The FITS are large, national cross sectional surveys that examine in-depth dietary behaviors of infants and children up to 48 months of age in the US [19]. After obtaining informed consent, dietary intake data were collected via telephone by trained interviewers using the multiple-pass 24 h recall methodology and nutrient intakes calculated using a food and nutrient composition database developed by the Nutrition Coordinating Center at the University of Minnesota (*n* = 3235) [19, 21]. A random subset of caregivers (*n* = 799) completed a second 24 h dietary recall to estimate usual nutrient intake distributions for the entire FITS sample. Daily volume of breastmilk was assumed to be 600 ml for breastfed infants, which is based on human milk consumption studies among 6–12 month old infants and is also the volume utilized by the Institute of Medicine (IOM) for establishing the Dietary Reference Intakes in this age group [22–25]. For mixed fed infants, the daily volume of breastmilk was calculated by subtracting the volume of infant formula or other milk from the 600 ml, also consistent with the IOM and NHANES methodology [22, 26]. The nutrient profile of human milk used in this analysis is aligned with that in the USDA standard reference [27, 28]. All study methods and procedures were approved by Institutional Review Boards at RTI International, University of Minnesota, Docking Institute of Public Affairs, and Fort Hays State University [19].

For this analysis, only infants aged 6–11.9 months old who were either breastfed (no infant formula provided, *n* = 296) or mixed fed (breastmilk and infant formula provided, *n* = 102) were included. Infants who were exclusively fed formula (*n* = 448) or who received neither formula nor breastmilk (i.e. cow milk) (*n* = 56) on the day of the recall were excluded. Infants were categorized into one of the following four categories: breastfed cereal consumer, breastfed non-consumer, mixed fed cereal consumer, or mixed fed non-consumer, as reported by the child’s caregiver at the time of the 24 h recalls. Following the National Cancer Institute method, usual nutrient intakes were calculated with and without nutrient intakes from dietary supplements (vitamin and/or mineral supplements) included [29]. The key nutrients evaluated included iron, zinc, vitamin C, thiamin, riboflavin, niacin, vitamin B6, folate, vitamin B12, calcium, phosphorus, magnesium, vitamin A, potassium, choline, vitamin D, and vitamin E.

Characteristics including race, household income, caregiver education, participation in WIC, use of dietary supplements, and meat intake of infants in each category were extracted. Weighted mean intakes of usual energy intakes were calculated for each group. Sample weighting methods were applied to control for bias between the survey sample and the US population. The Dietary Reference Intakes have an established EAR for iron and zinc and an Adequate Intake (AI) level for all other micronutrients for infants aged 6–12 months [30, 31]. The EAR is the level of nutrient intake determined to be adequate to meet the needs of 50% of the population [32]. When there is insufficient data to establish an EAR, an AI level is provided which is an estimate of the amount needed to ensure nutrition adequacy in the population [32]. In accordance with the application of Dietary Reference Intakes to populations provided by the National Institutes of Health, the percent of a population below the EAR are considered at risk for having inadequate nutrient intakes for that nutrient [32]. Applying the same principal to an AI level would likely be an overestimate of the percentage of the population at risk of inadequate intakes; however, the percent of the population at/above the AI level are considered at low risk of having inadequate nutrient intakes [32]. Therefore, we calculated the percentage of infants with nutrient intakes below the EAR for iron (6.9 mg) and zinc (2.5 mg) and the percentage of infants with intakes at/above the AI for all other nutrients [32]. Sources of iron were ranked according to percent contribution to total iron intake.

Descriptive statistics were tabulated for the characteristics, and significant differences in characteristics between cereal consumers and non-consumers among breastfed and mixed fed infants were determined using unpaired T-tests. The mean energy intake and percentage of infants with micronutrient intakes below the EAR or above AI level were analyzed for statistically significant differences between cereal consumers and non-consumers among breastfed infants and mixed fed infants using unpaired T-tests. A Bonferroni-corrected *p* value of 0.002 was used to determine statistical significance. SAS (version 9, SAS Institute Inc.: Cary, NC) and SAS-callable SUDAAN® (version 11, RTI International: Research Triangle Park, NC) software was used for all statistical analyses.

## Results

Characteristics were similar across categories (Table 1). Among breastfed infants, significantly more cereal consumers use dietary supplements compared to non-consumers (*p* = 0.0006). Total daily energy intake was significantly higher in cereal consumers compared to non-consumers among breastfed infants but not mixed fed infants. Iron supplement use was low across all categories.

**Table 1:** Sample Characteristics and energy intakes of 6–11.9 month old infants according to breastfeeding status and infant cereal consumption^a^

Characteristic % (SE)	Breastfed Infants Cereal Consumer ***n*** = 106	Breastfed Infants Non-Consumer ***n*** = 190	Mixed Fed Infants Cereal consumer ***n*** = 61	Mixed Fed Infants Non-Consumer ***n*** = 41
Child’s Sex Male	57.6 (4.8)	54.2 (3.6)	52.5 (6.4)	65.9 (7.4)
Child First Born	37.8 (5.1)	30.9 (3.6)	45.6 (6.6)	37.8 (8.0)
Race				
Hispanic	9.4 (2.8)	13.2 (2.5)	10.0 (3.9)	17.5 (6.0)
Non-Hispanic White	78.3 (4.0)	76.7 (3.1)	58.3 (6.4)	72.5 (7.1)
Non-Hispanic Black	4.7 (2.1)	6.4 (1.8)	15.0 (4.6)	7.5 (4.2)
Non-Hispanic Other	7.6 (2.6)	3.7 (1.4)	16.7 (4.8)	2.5 (2.5)
Income (US Dollars)				
Under 10,000	1.9 (1.3)	4.2 (1.5)	6.6 (3.2)	2.4 (2.4)
10,000 to 19,999	9.4 (2.8)	6.3 (1.8)	6.6 (3.2)	2.4 (2.4)
20,000 to 34,999	12.3 (3.2)	13.7 (2.5)	16.4 (4.7)	14.6 (5.5)
35,000 to 49,999	18.9 (3.8)	20.5 (2.9)	23.0 (5.4)	24.4 (6.7)
50,000 to 74,999	28.3 (4.4)	20.5 (2.0)	21.3 (5.2)	14.6 (5.5)
75,000 to 99,999	14.2 (3.4)	20.5 (2.9)	13.1 (4.3)	29.3 (7.1)
100,000 to 149,999	12.3 (3.2)	10.5 (2.2)	9.8 (3.8)	4.9 (3.4)
150,000 or more	2.8 (1.6)	3.7 (1.4)	3.3 (2.3)	7.3 (4.1)
Maternal Education				
High school or less	11.3 (3.1)	15.9 (2.7)	18.0 (4.9)	15.0 (5.7)
Some post-secondary	24.5 (4.2)	21.7 (3.0)	18.0 (4.9)	22.5 (6.6)
College or graduate	64.2 (4.7)	62.4 (3.5)	63.9 (6.1)	62.5 (7.7)
WIC Participant	30.2 (4.5)	23.2 (3.1)	39.3 (6.3)	24.4 (6.7)
Meat Intake^a^				
Non-Baby Food	27.4 (4.3)	33.7 (3.4)	9.8 (3.8)	31.7 (7.3)
Baby Food	6.6 (2.4)	4.7 (1.5)	4.9 (2.8)	0.0 (0.0)
Supplement Use^c^				
Any Supplement	37.7 (4.7)^a^	19.0 (2.8)^a^	27.9 (5.7)	22.0 (6.5)
Supplement with Iron	5.7 (2.3)	3.2 (1.3)	4.9 (2.8)	0.0 (0.0)
Mean Formula Intake	n/a	n/a	440.5 (40.0)	447.0 (54.1)
Total Calorie Intake^d^	819.43 (17.74)**	674.81 (11.30)**	763.04 (20.54)	720.16 (23.21)

*SE* Standard Error, *WIC* Women, Infants & Children

^a^All characteristics are unweighted estimates with the exception of total calorie intake

^a^Baby Food Meat = All commercially and homemade pureed meat excluding meats in pureed mixed dishes, Non-Baby Food Meat = All other forms of meat excluding meats in mixed dishes.

^c^Any supplement includes any vitamin/mineral supplement including those with iron

^d^Reported as weighted means. Calorie needs for 6–12 month old infants range from 505 kcal/day for 6 month old girls to 1000 kcal/day for 12 month old boys [33].

**p < 0.002

**Fig. 1:**
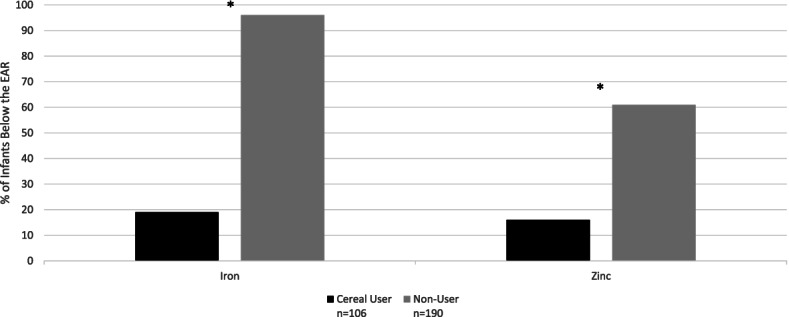
Percentage of breastfed cereal users and non-users aged 6–11.9 months with iron and zinc intakes below the Estimated Average Requirement including intakes from dietary supplements. * = *p* < 0.0001 comparing percent of cereal users and non-users below the Estimated Average Requirement (A Bonferroni-corrected *p* value of 0.002 was used to determine statistical significance)

When nutrient intakes of dietary supplements were included, 96% of breastfed non-consumers had iron intakes below the EAR (Fig. 1). This was significantly higher than the 19% of breastfed cereal consumers who had intakes below the EAR (*p* < 0.0001). Similarly, significantly more breastfed non-consumers had intakes below the EAR for zinc (61%) compared to breastfed cereal consumers (16%, *p* < 0.0001). When nutrient intakes of dietary supplements were excluded (not shown), the percent of breastfed cereal consumers and non-consumers with zinc intakes below the EAR remained the same (16 and 61% respectively, *p* < 0.0001), while iron intakes below the EAR changed slightly (19 and 98% respectively, *p* < 0.0001).

**Fig. 2:**
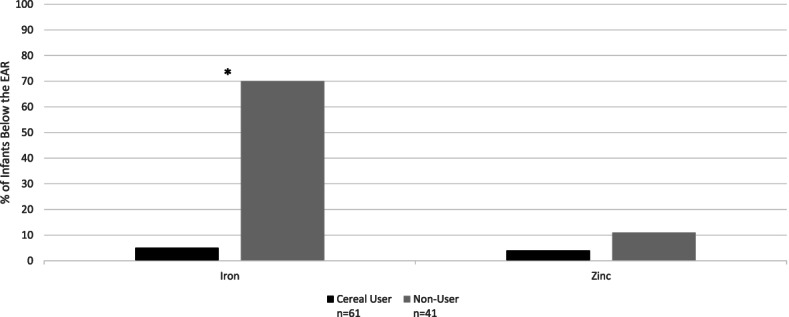
Percentage of mixed fed cereal users and non-users aged 6–11.9 months with iron and zinc intakes below the Estimated Average Requirement including intakes from dietary supplements. * = *p* < 0.0001 comparing percent of cereal users and non-users below the Estimated Average Requirement (A Bonferroni-corrected *p* value of 0.002 was used to determine statistical significance)

When nutrient intakes of supplements were included, the percentage of mixed fed non-consumers with iron intakes below the EAR was significantly higher than mixed fed cereal consumers (70% vs 5% respectively, *p* < 0.0001) (Fig. 2). The percentage of mixed fed cereal consumers and non-consumers with zinc intakes below the EARs were not significantly different (4% vs 11% respectively, *p* = 0.15). When nutrient intakes of supplements were excluded (not shown), only very slight differences in percentage of mixed fed cereal consumers and non-consumers below the EAR for iron (6% vs. 70% respectively, *p* < 0.0001) and zinc (4% vs. 11% respectively, *p* = 0.1563) were observed.

**Fig. 3:**
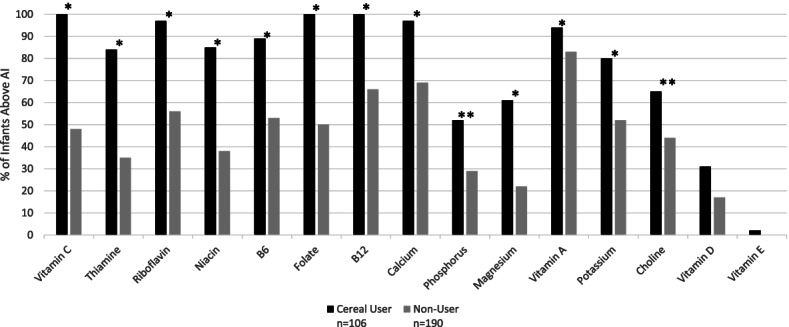
Percentage of breastfed infants (6–11.9 Months) with nutrient intakes above the Adequate Intake levels. * = *p* < 0.0001, ** = *p* < 0.002 comparing percent of cereal users and non-users above the Adequate Intake (A Bonferroni-corrected *p* value of 0.002 was used to determine statistical significance)

When nutrient intakes from supplements were included, a significantly higher percentage of breastfed cereal consumers had intakes above the AI level for 12 nutrients compared to non-consumers with the exception of Vitamin D and Vitamin E (Fig. 3). When nutrient intakes from dietary supplements were excluded, these differences did not change (not shown).

**Fig. 4:**
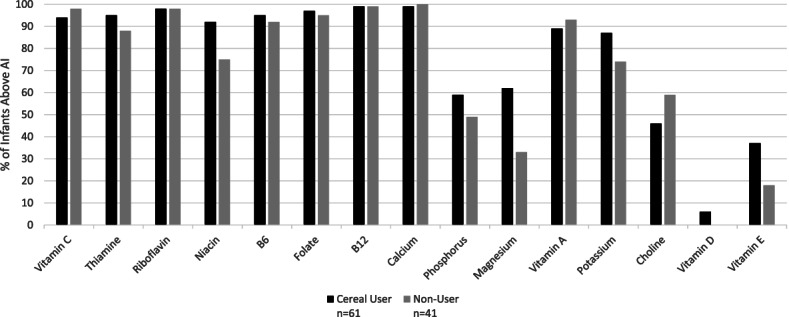
Percentage of mixed fed infants (6–11.9 Months) with nutrient intakes above the Adequate Intake levels. (A Bonferroni-corrected *p* value of 0.002 was used to determine statistical significance)

Among mixed fed infants, there were no significant differences in the percentage of infants with nutrient intakes above the AI levels between cereal consumers and non-consumers with or without nutrient intakes from supplements included (Fig. 4; data without supplements not shown).

Grains were a top source of iron among breastfed cereal consumers and non-consumers (Table 2). Among breastfed infants who did not consume infant cereal, family cereals (non-infant cereals including ready-to-eat and hot cereals) were the top source of iron. Among mixed fed cereal consumers, infant cereal outranked infant formula as the top source of iron. Meats contributed less than 2% of total iron intake in all groups except for breastfed non-consumers where meat contributed 6% of total iron intake, mainly from chicken and turkey.

**Table 2:** Ranked Sources of Iron among breastfed and mixed fed cereal consumers and non-consumers (6–11.9 Months)^a^

Rank	Breastfed				Mixed Fed			
	Cereal Consumer ***n*** = 106 Mean Iron Intake = 10.6 mg		Non-Consumer ***n*** = 190 Mean Iron Intake = 2.3 mg		Cereal Consumer ***n*** = 61 Mean Iron Intake = 14.1 mg		Non-Consumer ***n*** = 41 Mean Iron Intake = 6.1 mg	
	Iron Source	%	Iron Source	%	Iron Source	%	Iron Source	%
**1**	**Grains**	**83**	**Grains**	**43**	**Grains**	**59**	**Milk**	**74**
	Infant Cereal	75	Family Cereal	23	Infant Cereal	54	Infant Formula	72
	Family Cereal	4	Baby Grain FF	8	Baby Grain FF	2	Human Milk	2
	Baby Grain FF	2	*Puffs*	*5*	Family Cereal	2		
			*Crackers*	*3*				
			Pancake/Waffle	4				
			Pasta/Rice	3				
			Bread/Rolls	2				
			Crackers/Pretzels	2				
**2**	**Vegetables**	**5**	**Vegetables**	**13**	**Milk**	**33**	**Vegetables**	**7**
	Baby Vegetables	3	Baby Vegetables	7	Infant Formula	*31*	Baby Vegetables	5
	Vegetables	2	Vegetables	6			*Mixed Vegetable*	*3*
**3**	**Fruit**	**4**	**Meat/Proteins**	**12**	**Vegetables**	**4**	**Grains**	**7**
	Baby Fruit	2	Meats	6	Baby Vegetables	3	Baby Grain FF	3
			*Chicken/Turkey*	*3*			*Puffs*	*3*
			Non-Meat Protein	6				
			*Eggs*	*3*				
			*Beans/Legumes*	*2*				
**4**	**Meat/Proteins**	**2**	**Fruit**	**10**	**Fruit**	**3**	**Fruit**	**4**
			Baby Fruit	6	Baby Fruit	2	Baby Fruit	3
			*Apples/apple mix* Fruit	*3* 4				
**5**	**Mixed Dishes**	**2**	**Milk** Human Milk	**9** 7	**Meat/Proteins**	**1**	**Meat/Proteins**	**4**

*FF* Finger Foods

^a^May not add up to 100% because table only includes sources of iron contributing at least 2% of iron to the total intake

## Discussion

In this study, significantly fewer breastfed and mixed fed infants who consumed infant cereal had inadequate intakes of iron. When nutrient intakes from supplements were excluded, 98% of breastfed infants who did not consume infant cereal fell below the EAR for iron, compared to only 19% of breastfed cereal consumers. Also, breastfed cereal consumers were less likely to have inadequate intakes of zinc compared to non-consumers. Among breastfed infants, significantly more cereal consumers were at low risk of having inadequate nutrient intakes for 12 additional nutrients. Among mixed fed infants, supplemental formula provided adequate intakes of most nutrients, but mixed fed infants require an additional complementary source of iron to meet requirements.

Our findings that infant cereal contributes to nutrient intakes in infants are consistent with other studies. A NHANES analysis examining the food sources of energy and nutrients among infants and toddlers aged 0–24 months, found that cereal, including infant cereal, was the most important contributor to micronutrient intakes [34]. Similarly, another NHANES analysis which included formula fed and breastfed infants, compared infant cereal consumers to non-consumers aged 0–24 months and found that infant cereal consumers had higher intakes of iron, calcium, magnesium, zinc, and vitamin E compared to non-consumers. They also found that fewer cereal consumers fell below the recommended levels of iron, calcium, and vitamin E [35]. In this study, which differed from the NHANES studies in that it excluded fully formula fed infants and was focused on 6–11.9 month old infants, we found that cereal intake had an impact on a wider range of nutrients for breastfed infants, but not mixed fed infants. This is likely due to the nature of infant formulas being heavily fortified. We did not see significant differences in vitamin E intakes between groups. Of note, very few breastfed infants had vitamin E intakes above the AI, but this is likely an underestimate as the methodology used to set the AI included more recent estimate of the vitamin E content in human milk (4.9 mg/L) which is higher than the level in our database (0.82 mg/L) [36]. In their scientific report, the dietary guidelines advisory committee reported food modeling exercises for 6–12 month old breastfed infants where they found the feasibility of meeting nutrition needs without fortified foods would require further research [18]. This supports our hypothesis that infant cereal can play an especially important role among breastfed infants who are not supplemented with infant formula, but it is still an important source of iron and other essential nutrients for all infants regardless of feeding method.

We found that fortified infant and family cereals were the top food sources of iron. Family cereal (all cereal aside from infant cereal) was the top source of iron among breastfed infant non-consumers, and the second top source of iron among breastfed and mixed fed cereal consumers. A recent NHANES analysis of children aged 6 months to 17 years found that those who ate ready-to-eat cereal (not including infant cereal or hot cereals) had higher micronutrient intakes, including iron, higher whole grain intake, and better diet quality compared to those who did not eat cereal [37]. Although ready to eat cereal is an important source of iron and other key nutrients, it is not a replacement for infant cereal for this age group as it has less iron per gram of cereal. The iron in 15 g (1/4 cup) of infant cereal (6.75 mg) is roughly equivalent to the amount of iron in 23 g (3/4 cup) of toasted oat cereal (7.4 mg), and the bioavailability does not vary greatly [27].

We also found that few infants consumed baby food meat (intake ranged from 0% of mixed fed, non-consumers, to 6.6% of breastfed cereal consumers). More non-consumers ate non-baby food meats compared to cereal consumers (33.7% 4vs. 27.4% among breastfed infants and 31.7% vs. 9.8% among mixed fed infants respectively). This is consistent with our previous findings that cereal consumers were also more likely to consume baby food meat [3]. In the US, overall meat intake is very limited among infants and toddlers, and when meat is consumed, it is typically not an iron rich source [34]. For example, the NHANES 2005–2012 data indicated that meats contributed only 1.4 and 8.3% to the total dietary iron intake among 6–11.9 month old infants and 12–23.9 month old toddlers respectively [34]. This is similar to our findings that chicken or turkey were the most commonly consumed meats and contributed only a small amount to total iron intake.

Despite the AAP recommendations for breastfed infants to receive iron and vitamin D supplements, dietary supplement use was low in the overall FITS survey among infants 6–11.9 months old with only 15% consuming any type of supplement and < 5% consuming an iron supplement [38]. Our findings indicate that although supplement use was higher in our sample of infants receiving breastmilk compared to the overall FITS population, caregivers who provided infant cereal were also more likely to provide a dietary supplement compared to those who did not (37.7% vs. 18.9% among breastfed infants and 27.9%vs. 22.0% among mixed fed infants respectively), but iron supplement use was very low (ranged from 0.0% of mixed fed non-consumers to 5.7% of breastfed cereal consumers).

The exact causes of declining infant cereal use are not entirely known, but it is likely that several factors contributed. Historically, infant rice cereal was widely recommended as a first food for babies, starting at around 4 months of age [39]. More recently, it is recommended that complementary feeding begin at approximately 6 months of age and the order of food introduction is less important [15, 39]. Another factor may be concerns over the arsenic content of rice cereal. In 2016, the Food and Drug Administration (FDA) issued an action level for inorganic arsenic in infant rice cereal, but recent analyses indicated that potential arsenic exposure from infant rice cereal is declining [40, 41]. The AAP maintains that rice cereal is safe, but that infants should be offered a variety of infant cereals including those made with oats, wheat or multi-grains [15, 42]. Finally, there may be a perception that iron gaps may be less of a concern due to infant cereal fortification and the WIC program, leading to iron becoming less of a focus in nutrition guidance by healthcare providers.

The FITS is the largest study of feeding practices and nutrient intakes among infants and toddlers in the US. This is the only analysis to date that specifically explores the role of infant cereal in the diets of breastfed infants. There are, however, several limitations to this study. Although dietary information was reported by caregivers most likely to know the child’s food consumption, self-reported dietary intake studies can over- or under-report actual food consumption. Also, though established methods for estimating breastmilk intake were used, it is only an estimate [22]. The USDA breastmilk composition data was used, which is based on older studies from a small sample of women [28]. We limited our focus to micronutrients and did not assess differences in macronutrient intakes. Finally, we did not explore how other complementary food sources, such as meat, may impact iron and other nutrient intakes in this population.

## Conclusions

Complementary feeding practices are changing in the US. In recent years, use of iron fortified infant cereal has declined, infants eat little iron rich meat and dietary supplement use is also low. This is especially concerning for the growing population of exclusively breastfed infants who rely on complementary foods and supplementation for adequate iron intake after the iron stores from birth are depleted around four to 6 months of age [15]. National surveillance of iron deficiency among infants should be strongly considered. The main conclusion of this study is that the source of iron in this population is primarily from infant cereal, infant formula, or a combination of both. These findings should serve as evidence that iron fortified infant cereal is an important component of the infant diet. If arsenic exposure is a concern, non-rice infant cereals can be recommended.

In conclusion, when introducing complementary foods, it is not just the “when”, but also the “what” that is important. As pediatricians continue to advise parents on the introduction of complementary foods at around 6 months of age, it is imperative that the importance of fortified infant cereals and iron rich meat in the breastfed and mixed fed baby’s diet is emphasized.

## Data Availability

The dataset analyzed in the current study is proprietary and not publicly available. Please direct any inquiries regarding the data used in this manuscript to the corresponding author.

## Abbreviations

AAP: American Academy of Pediatrics
AI: Adequate Intake
FDA: Food and Drug Administration
EAR: Estimated Average Requirement
FITS: Feeding Infants and Toddlers Study
IOM: Institute of Medicine/National Academy of Medicine
NDSR: Nutrition Data System for Research
NHANES: National Health and Nutrition Examination Survey
USDA: United States Department of Agriculture
WIC: Supplemental Nutrition Program for Women, Infants, and Children
